# Deciphering the keystone position of abundant species within surface-dwelling microbial aggregates in paddy soils

**DOI:** 10.1128/aem.01399-25

**Published:** 2025-11-11

**Authors:** Danfeng Jin, Hua Hu, Chen Zhou, Nianhua Tang, Lingjia Liu, Eleonora Silvano, Yin Chen, Pengfei Sun

**Affiliations:** 1 College of Ecology and Environment, Yuzhang Normal University 499372, Nanchang, China; 2 Jiangxi Yijie Environmental Protection Technology Co., Ltd, Nanchang, China; 3 State Key Laboratory of Soil and Sustainable Agriculture, Institute of Soil Science, Chinese Academy of Sciences 74586 , Nanjing, China; 4 School of Life Sciences, University of Warwick 117213, Coventry, United Kingdom; 5 School of Biosciences, The University of Birmingham 85442, Birmingham, United Kingdom; 6 University of Chinese Academy of Sciences, Nanjing, China; University of Georgia Center for Food Safety, Griffin, Georgia, USA

**Keywords:** microbial aggregates, microbial cultivation system, abundant subcommunity, keystone species, paddy fields, periphyton

## Abstract

The surface soil horizon, functioning as the biogeochemical nexus in paddy ecosystems, harbors architecturally complex microbial consortia where abundant and rare taxa exhibit functional redundancy. While these aggregates drive critical nutrient cycling processes, the mechanistic partitioning of ecological roles between abundant and rare subcommunities remains obscured, limiting the development of microbiota-targeted agricultural optimization strategies. To experimentally dissect their functional hierarchies, we developed a controlled culturing system (26°C, 12 h light–dark cycle, 10,000–12,000 lux; nutrient supply: 10% soil leachate + 0.5% mineral solution) to selectively suppress rare taxa while preserving the abundant taxa. Systematic functional partitioning revealed three cardinal determinants of abundant subcommunity ecological predominance: abundant taxa (i) account for >50% of the microbial diversity; (ii) dominate community assembly processes; and (iii) exert greater influence on carbon, nitrogen, and sulfur cycling compared to rare taxa. Our approach establishes causal relationships beyond bioinformatic speculation, providing a functional disentanglement framework that redefines abundant taxa as keystone engineers of aggregate stability and functionality. This conceptual shift holds significant promise for the advancement of precision agriculture and the development of more sustainable nutrient management approaches.

**IMPORTANCE:**

Moving beyond traditional approaches to bioinformation analysis, this study employed an experimental strategy featuring a novel microbial filtration system. This system was designed to selectively remove rare species, thereby enabling the identification of the predominant roles played by abundant species within microbial aggregates. The findings demonstrate that abundant species are critical for maintaining community stability, governing assembly processes, and exerting greater ecological functions. Beyond introducing a filtration technique capable of distinguishing abundant and rare species in periphyton-like microbial communities, this work provides experimental evidence supporting the prioritization of abundant species in future efforts aimed at regulating periphyton growth or developing periphyton-based biotechnologies for nutrient cycling optimization.

## INTRODUCTION

Microbes in surface soil layer play pivotal roles in shifting nutrient cycling and ecosystem functioning (1–3). These surface-dwelling microbes, often organized as microbial aggregates, demonstrate remarkable potential for nutrient retention and turnover, making them crucial targets for developing sustainable agricultural biotechnologies (4, 5). Among these aggregates, periphyton represents a unique and ecologically significant microecosystem that substantially influences elemental cycling (carbon, nitrogen, phosphorus, iron, manganese, etc.) in paddy fields (6–11).

The ecological significance of periphyton lies in its taxonomically and functionally diverse assemblages, which emerge as a promising tool for sustainable rice production (8). While according to the literature, abundant taxa typically dominate biomass and core nitrogen cycling processes, rare taxa are increasingly recognized as critical contributors to community resilience and functional redundancy (12–14). Recent studies further indicate that both abundant and rare taxa can be important for ecosystem multifunctionality, but their relative contributions remain unresolved across ecosystems (15–17).

The prevailing comprehension of the dynamics within microbial communities predominantly stems from bioinformatics analyses. Although these analyses offer substantial value, they might fall short in comprehensively representing the intricate ecological interactions that occur in their natural environment (15–17). Thus, there is an urgent imperative for experimental approaches capable of mechanistically disentangling the distinct contributions of abundant versus rare taxa to community assembly and ecosystem functioning.

To experimentally disentangle the ecological significance of abundant versus rare taxa, we designed an integrated experimental framework centered around a novel three-level, three-factor Box-Behnken design (BBD)-based cultivation system for targeted enrichment of abundant subcommunities of periphyton. For example, previous studies have shown that abundant taxa often dominate biomass production and nitrogen cycling (16), whereas rare taxa may contribute disproportionately under stress conditions by supporting complementary functions (15–17). These contrasting roles highlight the need for an experimental system that can separate and directly test the contributions of abundant and rare taxa. This system enabled the establishment of abundant species-dominated assemblages while maintaining key ecological functions observed in natural environments. Through comparative analysis of cultivated (abundant species-enriched) and *in situ* periphyton communities using results of high-throughput sequencing and quantitative PCR (qPCR) gene chips, we identified keystone species within the periphyton community based on their contributions to community structure, diversity maintenance, and assembly processes. Our study advances microbial ecology research through two key innovations: (i) the developed cultivation system providing a methodological breakthrough for differentiating functional contributions of microbial subpopulations, and (ii) present empirical evidence establishing abundant species as keystone taxa in periphyton communities, demonstrating their dominance in community assembly (>80% contribution to prokaryotic diversity) and biogeochemical cycling (particularly carbon, nitrogen, and sulfur transformations). Our approach establishes causal relationships beyond bioinformatic speculation, providing a functional disentanglement framework that redefines abundant taxa as keystone engineers of aggregate stability and functionality. This conceptual shift holds significant promise for the advancement of precision agriculture and the development of more sustainable nutrient management approaches.

## MATERIALS AND METHODS

### Periphyton collection from paddy fields

A paddy field located in the subtropical zone (31°58′12″ N, 119°21′ E) was selected as the sampling site for periphyton collection due to periphyton in subtropical areas generally exhibiting higher species richness (8). Three sampling sites were randomly distributed within every 200 m^2^ of the paddy field using a block distribution method. Periphyton samples were collected 7–15 days after rice transplanting, specifically from paddy fields where periphyton growth was robust (7). Periphyton samples were obtained by scraping from the soil surface, following established protocols (7, 18). Samples were transported to the laboratory on ice packs and stored at −20°C until further analysis or as the control.

### Preparation of soil extraction

To prepare the soil extract, soil samples were collected from paddy fields and dried at 105°C–115°C. Subsequently, 52 g of the dried soil sample was placed into a 50 mL container, followed by the addition of 25 mL of a 0.5 M carbonate extraction solution (19–21). The soil was stirred thoroughly using a glass rod, and then a half spatula of activated charcoal was introduced into the sample. The mixture was vigorously shaken for 1–2 minutes and allowed to settle for 5–10 minutes. The resulting solution was filtered to obtain the soil leachate.

### Construction of microbial filtration system

Three ecological factors were identified as critical for periphyton cultivation: soil extract, mineral solution, and culture temperature. Soil extract was included to approximate the nutrient profile of paddy soils, while the mineral solution supplied essential inorganic nutrients; culture temperature was selected as a key driver of periphyton development under controlled conditions (8). To optimize these factors, we applied a three-level, three-factor BBD within response surface methodology (RSM) (22, 23). In this study, the term “microbial filtration system” refers to culture-based filtering achieved by adjusting medium composition and temperature so that abundant taxa are retained, whereas rare taxa decline during cultivation, rather than to any physical filtration device. The mineral solution followed Woods Hole (WC) medium (24, 25).

A total of 17 runs were conducted with factors tested at three coded levels (−1, 0, and + 1) (Table 1). Abundant species richness was chosen as the response variable (*Y*) because preserving the diversity/stability of abundant taxa was the benchmark for successful cultivation, while rare taxa were selectively reduced. The second-order polynomial used for model fitting was:


(1) $$Y=\beta_{0}+\sum \beta_{i}X_{i}+\sum \beta_{ii}X^{2}+\beta_{ij}X_{i}X_{j}$$

**TABLE 1:** Coding and levels of experiment factors

Factor	Symbol	Code level		
		−1	0	1
Soil extract (% in volume)	*X*_1_	0	10	20
Mineral solution (%)	*X*_2_	0.5	1.0	1.5
Culture temperature (℃)	*X*_3_	26	30.5	35.0

where *Y* is the predicted response; *β*_0_ is the intercept; *β*_*i*_ is the linear coefficient; *β*_*ii*_ is the quadratic coefficient; *β*_*ij*_ is the interaction coefficient between factors *X_i_* and *X_j_*; and *X_i_* and *X_j_* are input variables, which are shown in Table 1 (26). The optimized recipe thus provides nutrient conditions that favor abundant taxa and, through cultivation, effectively reduce the relative representation of rare taxa.

### Cultivation of the abundant species-dominated periphytons

Optimal dosages of soil extract and mineral solution, as well as culture temperature, were determined from the BBD model and used to prepare the cultivation medium. Five grams of freshly collected periphyton was inoculated into the optimized medium and incubated under greenhouse conditions (26°C; light intensity 10,000-12,000 lux; 12 h light–dark). After 10 days, cultivated periphyton was harvested for downstream analyses; *in situ* periphyton served as the control. The optimal condition was defined as the treatment preserving the highest diversity/stability of abundant taxa, ensuring ecological representativeness, while rare taxa were selectively reduced during cultivation (16).

### High-throughput sequencing of the periphytons

The total DNA was extracted from 2 g of wet biomass. This biomass was sourced from two different origins: periphyton that had been cultivated using a microbial cultivation system and samples collected directly from paddy fields. The extraction process employed the E.Z.N.A. Water DNA Kit (D5525-02, manufactured by Omega Bio-tek, USA). Subsequently, the 16S rRNA and 18S rRNA genes were amplified via PCR using primers 515F (5′-GTGCCAGCMGCCGCGGTAA-3′)/907R (5′-CCGTCAATTCMTTTRAGTTT-3′) and 528F (5′-GCGGTAATTCCAGCTCCAA-3′)/706R (5′-AATCCRAGAATTTCACCTCT-3′) (7, 18), respectively. The integrity and purity of DNA were assessed by 1% agarose gel electrophoresis, while DNA concentration and purity were determined using microspectrophotometry (Bei Jing Kai Ao K5600, China). PCR products were purified using the E.Z.N.A. Gel Extraction Kit, and target DNA fragments were eluted with Tris-EDTA buffer. Library preparation followed the standard protocol of the NEBNext Ultra II DNA Library Prep Kit for Illumina, and sequencing was conducted on the HiSeq 2500 platform to obtain high-throughput data. Raw reads underwent quality filtering using the Cutadapt tool (v.1.9.1) to obtain high-quality clean reads. The UCHIME algorithm was employed to detect and remove chimeric sequences, ensuring the reliability of the obtained clean reads (27). Sequence analysis utilized UPARSE software, clustering sequences with ≥97% similarity into operational taxonomic units (OTUs) (28). Taxonomic annotation of OTUs was performed using the SILVA 138 database (https://www.arb-silva.de/).

### High-throughput qPCR gene chip

Both the periphyton cultivated using the microbial cultivation system and the samples collected from paddy fields were divided into five equal parts. From each part, 1 g of the sample was taken for high-throughput qPCR analysis using the Wafergen Smart Chip Real-Time qPCR platform. This platform was utilized to quantify the abundance of genes associated with carbon, nitrogen, phosphorus, and sulfur cycling. Three replicates for each sample were then prepared, resulting in a total of 15 samples from both the cultivated and collected periphyton for high-throughput qPCR gene chip analysis. Total DNA was extracted from each sample using the E.Z.N.A. Water DNA Kit (D5525-02, Omega Bio-tek), followed by purification and analysis. The qPCR was then performed, and fluorescence signals were detected using the SmartChip Real-Time PCR System. The detection status and cycle threshold (Ct) values for each gene in each sample were obtained using Canco software. Data were normalized to 16S rRNA as an internal reference to calculate the relative gene abundance. Additionally, absolute quantification of the 16S rRNA gene was performed using a Roche instrument. These data were then converted to obtain the absolute quantification of each gene. Quality control of the raw data were conducted based on the Ct values obtained from the SmartChip Real-Time PCR System and Canco software, following the methodology outlined in our previous work (7).

### Data and statistical analyses

#### OTU classification

OTUs use vegan in R for leveling to obtain high-quality clean readings for subsequent analysis. The abundance of high-quality OTUs was screened. Rare OTUs were defined as OTUs with a relative abundance being less than 0.01% in all samples, whereas abundant OTUs were defined as OTUs with a relative abundance being more than 1% (13). The α-diversity (richness, Shannon, Chao1, and ACE indexes) indexes were analyzed in R with “phyloseq,” “vegan,” “microbiome,” “picante,” and “adespatial” packages (29). α-Diversity was calculated with QIIME (v.1.9.1) and displayed with R software (30).

#### Neutral community model analysis

Neutral community model (NCM) analysis was used to predict the relationship between the detection occupancy frequency of abundant and rare OTUs and their relative abundance of the cultivated and *in situ* collected (from paddy fields) periphyton (12, 31). The analysis determined the importance of stochastic processes in the assembly of prokaryotic subcommunities. The 95% confidence intervals of each fitting statistic were calculated by bootstrapping with 1,000 bootstrap replicates. The parameters Nm and *R*^2^ represented estimates of dispersal between communities and the fit to the models, respectively. The computations were performed in R using the “picante,” “ape,” “rlang,” “ecodist,” “vegan,” “agricolae,” and “tidyverse” packages (8, 32).

#### Phylogeny-based community metrics analysis

To evaluate the phylogenetic community composition of cultivated and *in situ* collected (from paddy fields) periphyton, the beta nearest taxon index (βNTI) was calculated (33). If |βNTI| was more than 2, the determination process played an important role in the community composition, while if the value of |βNTI| was less than 2, the stochastic process would play an important role (34). Violin plots were employed to present the βNTI values of abundant species in the maintenance of assembly processes of prokaryotic and eukaryotic subcommunities of the cultivated and *in situ* collected periphytons.

#### Potential function analysis

To evaluate the contributions of abundant and rare species to the functional performance of periphyton, we employed high-throughput qPCR gene chip technology to quantify the absolute abundance of genes involved in carbon, nitrogen, phosphorus, and sulfur cycling in both cultivated and naturally collected periphyton (7, 35). The gene abundances in the naturally collected periphyton served as the baseline reference. We then calculated the proportion (%) of these functional genes in the cultivated periphyton relative to those in the naturally collected samples. This approach enabled us to assess the specific functional roles of the abundant taxa within the periphyton community.

#### Statistical analysis

Comparison of the mean proportion and difference in mean proportions of the top dominant abundant prokaryotic and eukaryotic species was performed using the statistical analysis of metagenomic profiles (STAMP) analysis. All statistical analysis was carried out using SPSS 16.0, Design Expert 10.0.1, or R 4.0.3, and the analysis results were presented using the “ggplot2” package of R to explore the ecological differences between *in situ* collected and cultivated periphytons (36). Other figures (such as high-throughput qPCR gene chip results) were prepared using OriginPro 2023b (OriginLab Corporation, Northampton, MA, USA). All statistical procedures (*t*-test) were conducted using SPSS software (v.16.0; SPSS Inc., Chicago, IL, USA).

## RESULTS

### Construction of the microbial cultivation system

A standard RSM, utilizing a three-level, three-factor BBD, was employed to optimize the dosage of each component in the microbial filtration system and to assess the impacts of the three factors (soil extract, mineral solution, and culture temperature) on the richness of abundant species in the cultivated periphytons in this study. The RSM yielded the following quadratic polynomial model (Eq. 2) for abundant species richness (*Y*):


(2) $$\begin{array}{c} Y=102.80-1.00\times X_{1}-14.62\times X_{2}-7.12\times X_{3}-3.00\times X_{1}\times X_{2}-2.00\\ \;\times X_{1}\times X_{3}+3.75\times X_{2}\times X_{3}-4.28\times X_{1}^{2}+10.47\times X_{2}^{2}+25.48\times X_{3}^{2} \end{array}$$

where *X*_₁_, *X*_₂_, and *X*_₃_ represent soil extract (%), mineral solution (%), and culture temperature (°C), respectively.

Variance analysis of the model (Table 2) confirmed its statistical significance (*P* < 0.05). The optimized system parameters included 10% soil leaching solution, 0.5% mineral solution, 26°C culture temperature, and 10,000–12,000 lux illumination under a 12 h–12 h light–dark cycle.

**TABLE 2:** Analysis of variance for the response surface quadratic model

Source	Sum of squares	DF	Mean square	*F* value	Prob >*F*
Model	5,577.48	9	619.72	3.52	0.0498
*X*_1_	8.00	1	8.00	0.045	0.8374
*X*_2_	1,711.13	1	1,711.13	9.71	0.0170
*X*_3_	406.13	1	406.13	2.30	0.1729
*X*_1_ *X*_2_	36.00	1	36.00	0.20	0.6650
*X*_1_ *X*_3_	16.00	1	16.00	0.091	0.7720
*X*_2_ *X*_3_	56.25	1	56.25	0.32	0.5898
*X*_1_^2^	76.95	1	76.95	0.44	0.5300
*X*_2_^2^	462.00	1	462.00	2.62	0.1495
*X*_3_^2^	2,732.53	1	2,732.53	15.50	0.0056
Residual	1,234.05	7	176.29		
Lack of fit	787.25	3	262.42	2.35	0.2137
Pure error	446.80	4	111.70		
Cor total	6,811.53	16			

The components of the mineral solution per liter of the medium with a volume ratio of 0.5% are NaNO_3_ 0.04255 mg·L^−1^, KH_2_PO_4_ 0.00436 mg·L^−1^, MgSO_4_·7H_2_O 0.01849 mg·L^−1^, NaHCO_3_ 0.0063 mg·L^−1^, CaCl_2_·2H_2_O 0.01838 mg·L^−1^, NaSiO_4_·9H_2_O 0.01421 mg·L^−1^, H_3_BO_3_ 0.012 mg·L^−1^, trace element solution I 0.0005 mL·L^−1^, VB_12_ solution 0.0005 mL·L^−1^, VB_1_ solution 0.0005 mL·L^−1^, and biotin solution 0.0005 mL·L^−1^. The composition of trace element solution I is Na_2_EDTA·2H_2_O 0.00218 g·L^−1^, FeCl_3_ 0.00158 g·L^−1^, ZnSO_4_·7H_2_O 0.011 g·L^−1^, MnCl_2_·4H_2_O 0.09 g·L^−1^, CuSO_4_·5H_2_O 0.00125 g·L^−1^, NaMoO_4_·2H_2_O 0.00315 g·L^−1^, and Na_3_VO_4_ 0.009 g·L^−1^. The composition of the VB_12_ solution is HEPES buffer 0.006 g·L^−1^ and VB_12_ 0.0000675 g·L^−1^. The composition of the VB_1_ solution is HEPES buffer 0.006 g·L^−1^ and VB_12_ 0.000168 g·L^−1^. The composition of the biotin solution is HEPES buffer 0.006 g·L^−1^ and biotin 0.0000125 g·L^−1^.

### Microbial cultivation system filters out rare species but keeps abundant species

**Fig 1:**
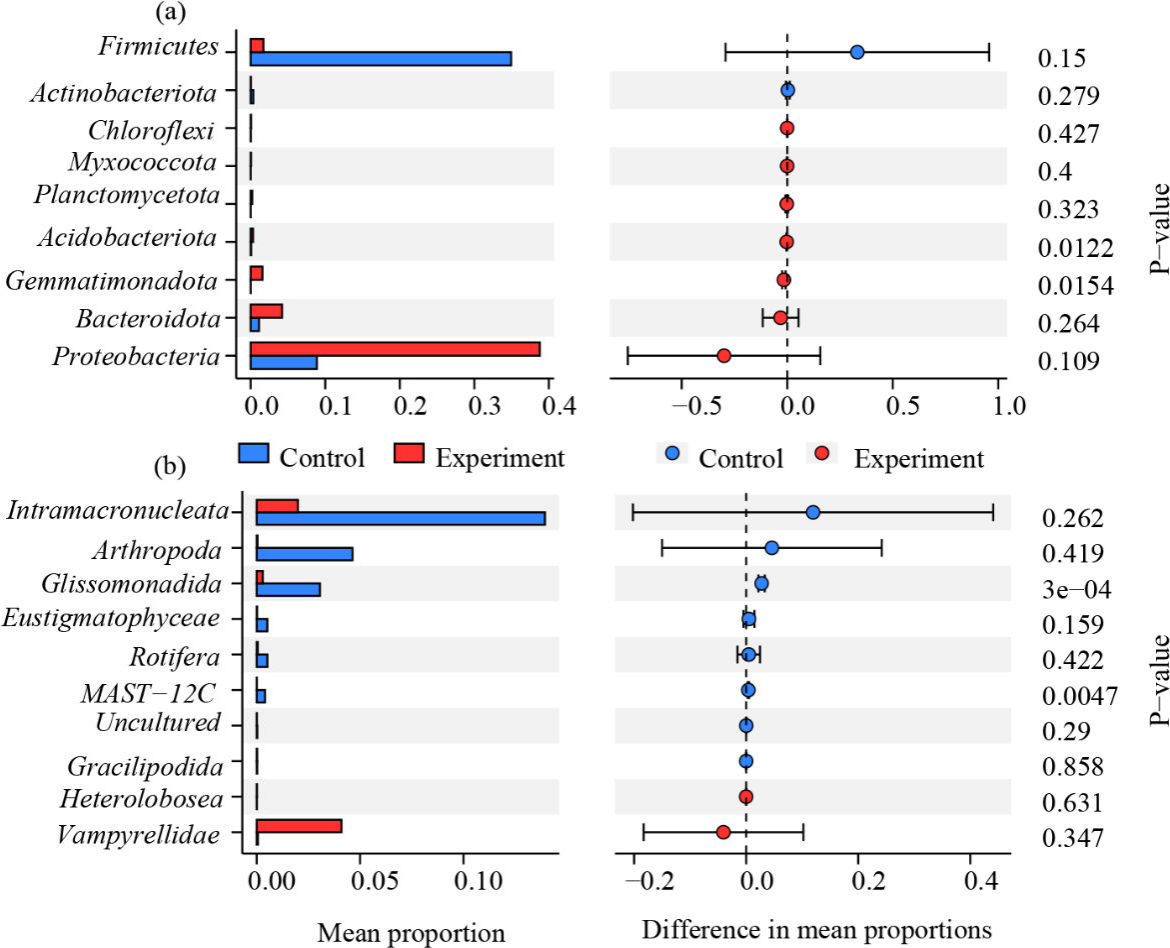
Comparison of the mean proportion and difference in mean proportions of the top nine dominant abundant prokaryotic and eukaryotic species (at phylum level, ≥1%) in the control (*in situ* collected periphytons) and experiment (cultivated periphytons). Panels **a** and **b** represent the prokaryotes and eukaryotes, respectively.

The microbial filtration system successfully replicated abundant communities, with both prokaryotic and eukaryotic communities in the cultivated periphytons closely resembling those in the *in situ* collected samples in terms of composition and abundance. For instance, prokaryotic subcommunities such as *Proteobacteria* and *Planctomycetota*, as well as eukaryotic subcommunities like *Vampyrellidae* and *Intramacronucleata*, consistently maintained their status as abundant species (relative abundances >1%) in both the cultivated and *in situ* collected periphyton (Fig. 1a). This suggests that the cultivation system effectively replicated these abundant communities. However, some significant differences were observed in the average proportions of *Firmicutes* and *Proteobacteria* among prokaryotes, and *Intramacronucleata*, *Arthropoda*, and *Vampyrellidae* among eukaryotes (*P* < 0.05, Fig. 1b).

Regarding rare subcommunities, the proportion of rare prokaryotic species decreased from 0.015% in *in situ* periphytons to 0.006% in cultivated ones, while rare eukaryotic species decreased from 0.007% to 0.001%. These results demonstrate the system’s efficacy in selectively filtering out rare species while maintaining abundant ones to establish an abundant species-dominated periphyton. Furthermore, the system reduced the proportion of uncultured prokaryotic and eukaryotic species in periphytons, declining from 0.116% to 0.105% and from 0.023% to 0.005%, respectively.

### Abundant species contribute differently to community structure and diversity maintenance

Comparison of the high-throughput sequencing results between cultivated and *in situ* collected periphytons revealed distinct contributions of abundant species to community structure and diversity. In the *in situ* collected periphytons, 3,319 prokaryotic and 882 eukaryotic OTUs were identified, whereas 775 prokaryotic and 247 eukaryotic high-quality OTUs were obtained from cultivated samples.

**Fig 2:**
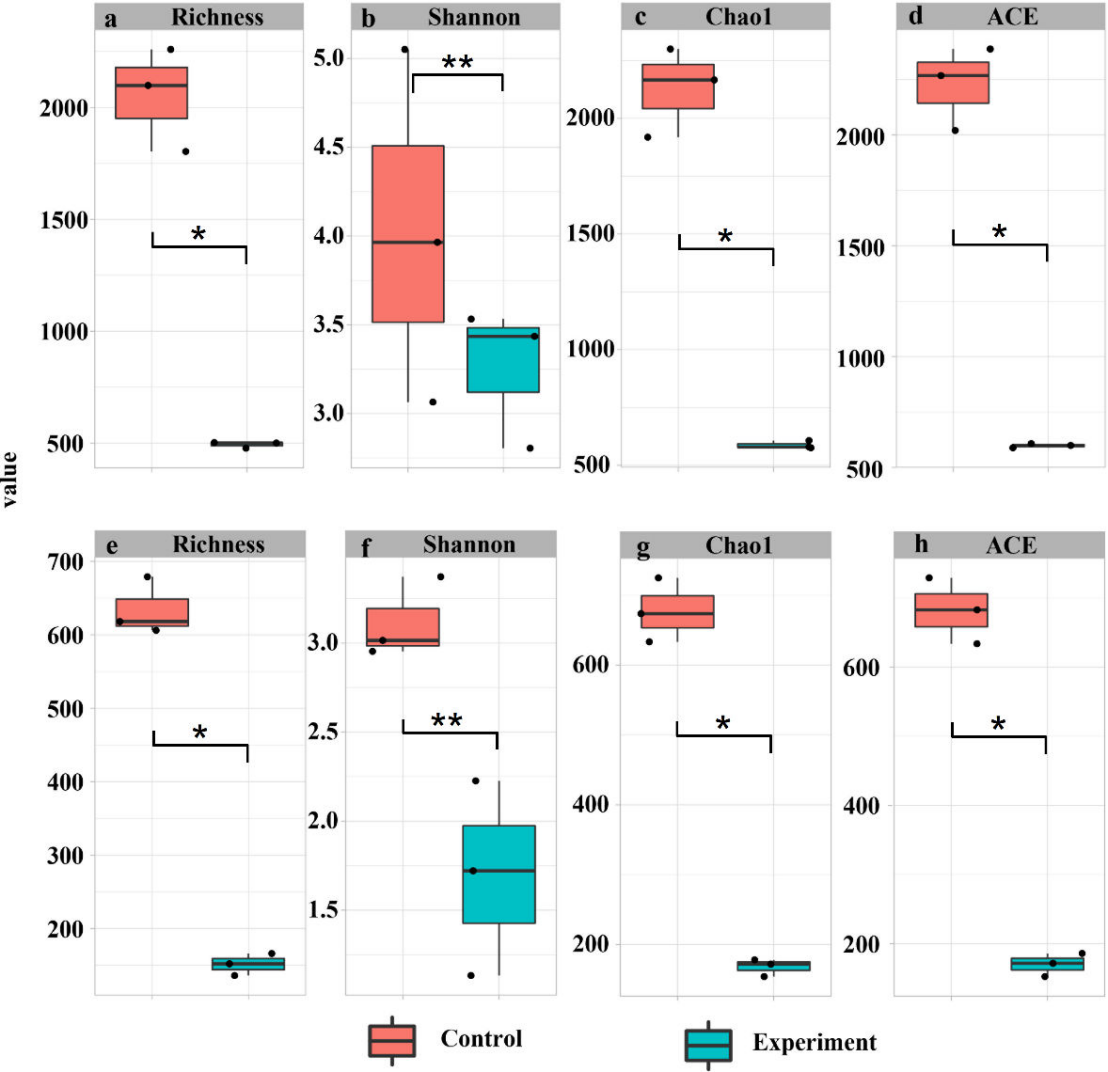
Indexes of α-diversity between cultivated (experiment) and *in situ* collected (control) periphytons. (**a–d**) Richness, Shannon, Chao1, and ACE indexes of prokaryotic communities, respectively. (**e–h**) Richness, Shannon, Chao1, and ACE indexes of eukaryotic communities, respectively. *P < 0.05, **P < 0.01.

Comparison of community richness (Chao1 and ACE, which are richness estimators that emphasize species counts) between *in situ* collected and cultivated periphytons showed that abundant species contributed less than 30% to overall structure in both prokaryotes and eukaryotes (*P* < 0.05, Fig. 2), indicating a predominant role of rare species in structure maintenance.

The Shannon index was used because it accounts for both richness and evenness, thereby reflecting integrated community diversity. Results showed that abundant prokaryotes accounted for over 80% of periphyton community diversity, while abundant eukaryotes contributed more than 50% (*P* < 0.05, Fig. 2). These findings highlight that while abundant species have minimal impact on periphyton structure (richness), they significantly contribute to maintaining diversity within periphyton communities.

### Roles of abundant species in assembly processes

**Fig 3:**
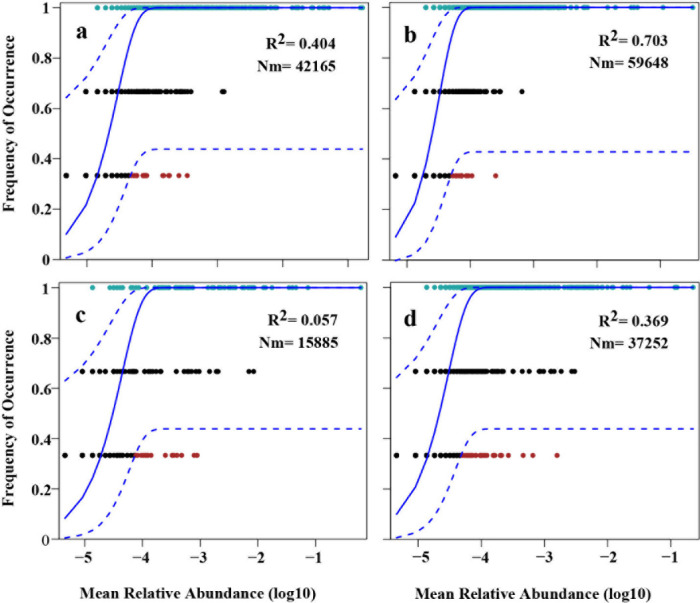
Fit of the neutral community model (NCM) of community assembly. The solid blue lines indicate the best fit to the NCM, and the dashed blue lines represent 95% confidence intervals around the model prediction. OTUs are shown in different colors. Nm represents the product of metacommunity size (n) and mobility (m), and *R*^2^ represents the overall goodness of fit of the model. **(a** and **b)** Prokaryotes of cultivated and *in situ* collected periphytons, respectively. **(c** and **d)** Eukaryotes in cultivated and *in situ* collected periphytons, respectively.

Null model and NCM analyses were employed to investigate the roles of abundant species in the assembly processes of prokaryotic and eukaryotic communities within the periphyton. The NCM analysis successfully estimated a significant portion of the relationship between the occurrence frequency of prokaryotic OTUs and their relative abundance variations, explaining 40.4% and 70.3% of the community variance in cultivated and *in situ* collected periphytons, respectively. The Nm was 42,165 for cultivated periphytons compared to 59,648 for *in situ* collected periphytons (Fig. 3a and b). For eukaryotic OTUs, the NCM analysis explained only 5.7% and 36.9% of the community variance in cultivated and *in situ* collected periphytons, respectively, with an Nm of 15,885 for cultivated periphytons versus 37,252 for *in situ* collected ones (Fig. 3c and d). Abundant prokaryotic and eukaryotic OTUs collectively contributed 58% and 15% to the explained variance in subcommunities by NCM, respectively.

**Fig 4:**
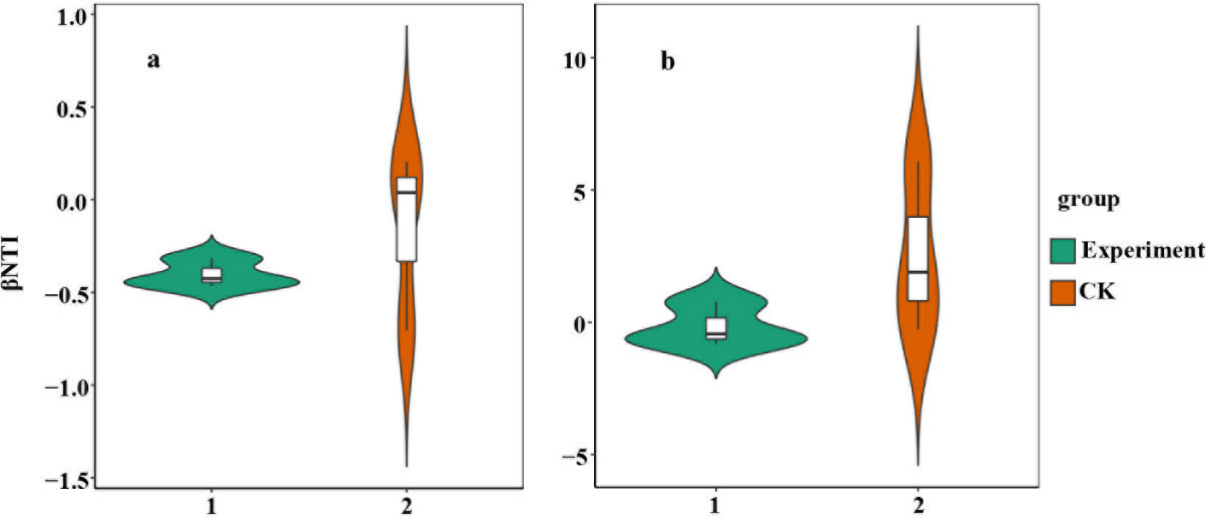
Contribution comparison of abundant species in the maintenance of assembly processes of prokaryotic (**a**) and eukaryotic (**b**) subcommunities of the cultivated (experiment) and *in situ* collected (CK) periphytons. A violin plot of βNTI values grouped by the prokaryotic (**a**) and eukaryotic (**b**) subcommunities of the periphyton.

Furthermore, βNTI values were calculated to explore the community assembly processes between cultivated and *in situ* collected periphytons. For prokaryotes (Fig. 4a), both cultivated and *in situ* collected periphytons exhibited βNTI values within the range of −2 to 2, suggesting that filtering out rare species to retain abundant species did not significantly alter the prokaryotic community assembly mechanism (*P* = 0.1). This underscores the predominant role of abundant prokaryotic species in the assembly processes of periphyton prokaryotic communities.

For eukaryotes (Fig. 4b), *in situ* collected periphytons displayed more dispersed βNTI values, whereas cultivated periphytons, which filtered out rare species, showed βNTI values more concentrated around 0. Despite this shift, the difference in βNTI values between cultivated and *in situ* collected periphyton was not statistically significant (*P* = 0.306). This indicates that there was no significant difference in the eukaryotic assembly process between the cultivated and *in situ* periphyton. Overall, these findings indicate that filtering out rare species did not significantly affect the assembly processes for either prokaryotic or eukaryotic communities, with abundant species continuing to dominate the assembly process in both cases.

### Abundant prokaryotes maintain the majority functions of periphyton

**Fig 5:**
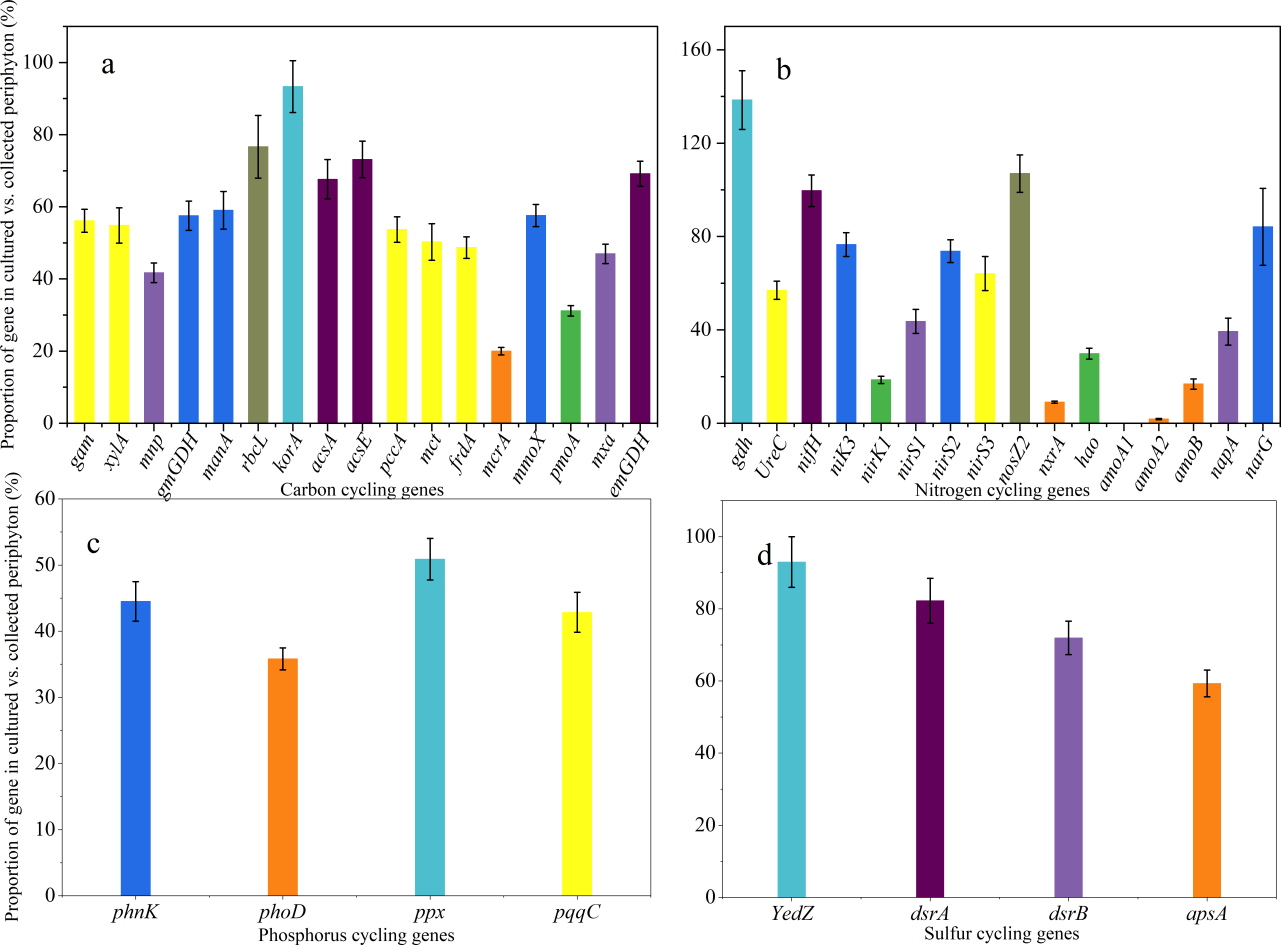
Roles of abundant prokaryotic communities in function maintenance. The roles were determined by calculating the proportion (%) of carbon (**a**), nitrogen (**b**), phosphorus (**c**), and sulfur (**d**) functional genes in the cultivated periphyton relative to those in the naturally collected samples.

High-throughput qPCR analysis revealed that abundant taxa in periphyton have a markedly greater impact on the regulation of carbon, nitrogen, and sulfur cycling than rare taxa. We identified 17 carbon cycling genes, including those involved in carbon degradation, fixation, and methane metabolism, in both cultured and naturally collected periphyton (Fig. 5a). Of these, 12 genes showed proportions exceeding 50%, with *korA* reaching the highest at over 93% ± 7%. The remaining five genes had proportions ranging from 20% ± 1% to 49% ± 3%. On average, the abundance of all carbon cycling genes in cultured periphyton relative to collected periphyton exceeded 56% ± 4%, highlighting the dominant role of abundant taxa in carbon cycling.

For nitrogen cycling, 16 genes were detected (Fig. 5b). Of these, seven genes had proportions over 50%, with *gdh* and *nosZ2* reaching 100%, and *nifH* reaching 99% ± 7%. On average, the abundance of all nitrogen cycling genes in cultured periphyton was over 54% ± 5% relative to the collected periphyton. These findings indicate that although abundant taxa significantly impact nitrogen cycling, the role of rare species in nitrogen cycling is more diverse.

In phosphorus cycling, the proportions of *phnK*, *phoD*, *ppx*, and *pqqC* genes in cultured periphyton relative to collected periphyton were 45% ± 3%, 36% ± 2%, 51% ± 3%, and 43% ± 3%, respectively (Fig. 5c). These results suggest that rare taxa play a more prominent role in phosphorus cycling. Regarding sulfur cycling, the average proportion of sulfur cycling gene abundance in cultured periphyton relative to collected periphyton exceeded 77% ± 5% (Fig. 5d). The results indicate that abundant taxa are crucial for sulfur reduction. This is evidenced by the higher proportions of *YedZ*, *dsrA*, *dsrB*, and *apsA* genes in cultured periphyton compared to the collected periphyton.

## DISCUSSION

This study developed a novel microbial cultivation system to elucidate the distinct roles of abundant and rare species. Our findings demonstrate that the developed system effectively maintains ecological characteristics and functions of periphyton while selectively enriching abundant species, thereby advancing *in vitro* cultivation techniques and enhancing our understanding of microbial community dynamics in agricultural ecosystems (37). Unlike conventional enrichment methods such as dilution-to-stimulation and dilution-to-extinction, which often indiscriminately reduce community diversity or primarily recover rare taxa (38, 39), our approach uniquely suppresses rare taxa while preserving abundant taxa and their functional roles. This selective retention ensures ecological representativeness and enables direct experimental validation of abundant taxa as keystone drivers in periphyton communities.

Compared with traditional direct sampling, which provides a snapshot of *in situ* community composition but cannot disentangle the roles of abundant and rare taxa, our cultivation system introduces a mechanistic perspective by selectively suppressing rare taxa. The consistent α-diversity and βNTI patterns between cultivated and *in situ* periphyton suggest that abundant taxa remain the main drivers of assembly processes, even when rare taxa are excluded. This indicates that the community assembly mechanisms are largely determined by abundant taxa, while rare taxa contribute primarily to structural richness rather than to assembly rules. By contrast, direct sampling approaches cannot reveal this mechanistic separation, underscoring the advantage of our system in bridging observational ecology with experimental validation (33, 40, 41).

The challenge of cultivating soil microorganisms has long been recognized, with traditional methods failing to culture >99% of bacterial species (42). Our system addressed this limitation by incorporating critical ecological factors, such as soil extract, which has been shown to enhance microbial cultivability (23). The successful replication of most prokaryotic and eukaryotic communities from *in situ* periphyton demonstrates the system’s efficiency in community maintenance. More importantly, this system offers a way to separate and discern the cultivation and functions of abundant species from those of rare species within microbial aggregates.

Our findings contribute to the ongoing debate regarding the ecological roles of abundant versus rare microbial taxa. While previous studies have primarily relied on bioinformatics analyses (15–17), our experimental approach provides robust evidence supporting the keystone role of abundant species in periphyton communities, consistent with recent findings that keystone taxa stabilize microbial networks and sustain functional diversity across ecosystems (43, 44). This is particularly evident in their dominant contributions to community diversity (>80% for prokaryotes and >50% for eukaryotes) and assembly processes, consistent with previous observations in various ecosystems (13, 45).

At the same time, we acknowledge that rare taxa can also serve as important drivers of ecosystem multifunctionality, particularly under environmental stress or resource limitation (46, 47). However, most of these conclusions are based on correlative analyses and statistical predictions rather than on experimental validation. In contrast, our cultivation system was explicitly designed to suppress rare taxa, thereby providing a direct test of whether abundant taxa are sufficient to sustain community functions. Thus, our results do not exclude potential contributions of rare taxa; rather, they demonstrate that abundant taxa can maintain community assembly and major biogeochemical functions. Future reciprocal experiments, in which abundant taxa are suppressed while rare taxa are retained, will be required to fully assess the functional roles of rare taxa.

The functional significance of abundant species was particularly pronounced in biogeochemical cycling processes. Our gene chip analyses revealed their predominant role in carbon, nitrogen, and sulfur cycling, with specific implications for paddy ecosystem functioning. For nitrogen cycling, abundant species demonstrated beneficial roles through biological nitrogen fixation (*nifH*) and greenhouse gas mitigation (*nosZ2*) (48, 49), contrasting with the potential nitrogen loss associated with rare species-dominated processes (*amoA1* and *amoA2*) (50). For carbon dynamics, the active participation of abundant species in both carbon fixation *(rbcL* and *korA*) and degradation (*xylA* and *manA*) processes suggests their crucial role in maintaining soil carbon levels (51–53). For sulfur cycling, the dominance of abundant species in sulfur reduction processes (*dsrA* and *dsrB*) highlights their importance in soil health maintenance and environmental protection (54). Importantly, this dominance in carbon, nitrogen, and sulfur cycling also points to clear translational potential, as these abundant taxa could be harnessed in microbial inoculants and soil management practices to enhance nutrient efficiency and sustain soil health, thereby providing a practical basis for sustainable rice agroecosystem management (55–57). While rare species appear to play a more significant role in phosphorus cycling, abundant species still contribute to phosphorus availability through polyphosphate degradation (*Ppx*) (58), underscoring their multifaceted functional importance.

Although our findings were derived from subtropical paddy fields, ecosystem context is likely to shape the relative importance of abundant versus rare taxa. In temperate regions with stronger seasonal variability, community assembly and functional contributions of abundant taxa may be more dynamic, while in arid systems, water limitation could reduce the resilience and cycling capacity of both abundant and rare groups (45, 59). Future multiecosystem comparisons will therefore be essential to generalize the functional framework established here (60).

Overall, our findings highlight the dominant role of abundant species in periphytons for maintaining community structure, stability, and assembly processes, as well as their significant contribution to the ecological functions of periphyton. These findings have significant implications for paddy ecosystem management and microbial biotechnology development. The demonstrated capability of our cultivation system to maintain functional microbial communities while selectively enriching abundant species opens new possibilities for (i) developing targeted microbial inoculants for improved nutrient management, (ii) enhancing carbon sequestration in agricultural soils, (iii) mitigating greenhouse gas emissions from paddy fields, and (iv) maintaining soil health through optimized microbial community engineering.

## Data Availability

All raw sequences from this study have been submitted to the National Center for Biotechnology Information Sequence Read Archive database under accession numbers PRJNA1358573 (prokaryotic 16S rRNA) and PX655717 to PX656671 (eukaryotic 18S rRNA). All requests for materials should be addressed to Pengfei Sun (pfsun@issas.ac.cn).

## AFTER EPUB

*[This article was published on 11 November 2025 with an incorrect accession number in the Data Availability statement. The accession numbers were updated in the current version, posted on 22 December 2025.]*
